# Plasma desmosine for prediction of outcomes after acute myocardial infarction

**DOI:** 10.3389/fcvm.2022.992388

**Published:** 2022-11-21

**Authors:** Kashan Ali, Muhammad Zubair Israr, Leong L. Ng, Ify Mordi, Chim C. Lang, Elena Kuzmanova, Jeffrey T-J Huang, Anna-Maria Choy

**Affiliations:** ^1^Division of Molecular and Clinical Medicine, School of Medicine, University of Dundee, Dundee, United Kingdom; ^2^Department of Cardiovascular Sciences, School of Medicine, College of Life Sciences, University of Leicester, Leicester, United Kingdom; ^3^National Institute for Health and Care Research (NIHR) Leicester Biomedical Research Centre, Leicester, United Kingdom; ^4^National Institute for Health and Care Research (NIHR) Leicester Cardiovascular Biomedical Research Unit, Leicester, United Kingdom; ^5^Division of Systems Medicine, School of Medicine, University of Dundee, Dundee, United Kingdom

**Keywords:** acute myocardial infarction, biomarkers, GRACE score, plasma desmosine, cardiovascular outcomes

## Abstract

**Background:**

Elastin degradation is implicated in the pathology of vulnerable plaque. Recent studies show promising results for plasma desmosine (pDES), an elastin-specific degradation product, as a marker of cardiovascular disease (CVD) outcomes. The aim of this study was to investigate the potential role of pDES as a marker of clinical outcome in patients with acute myocardial infarction (AMI).

**Materials and methods:**

In this case-control study, we studied 236 AMI patients: 79 patients who had death and/or myocardial infarction (MI) at 2 years, and 157 patients who did not have an event at 2 years. pDES was measured using a validated liquid chromatography-tandem mass spectrometry method. Association of pDES with adverse outcomes, and the incremental value of pDES to global registry of acute coronary events (GRACE) score for risk stratification was assessed.

**Results:**

pDES levels were elevated in patients with the composite outcome of death/MI at 2 years (*p* = 0.002). Logistic regression analyses showed pDES to be associated with death/MI at 2 years [Odds ratio (OR) 5.99 (95% CI 1.81–19.86) *p* = 0.003]. pDES remained a significant predictor of death/MI at 2 years even after adjustment for age, sex, history of CVD, revascularisation, blood pressure, medications on discharge, Troponin I, and NT-proBNP levels.[OR 5.60 (95% CI 1.04–30.04) *p* = 0.044]. In another multivariable model including adjustment for eGFR, pDES was significantly associated with the composite outcome at 6 months, but not at 2 years follow up. DES was also able to reclassify risk stratification for death/MI at 6 months, when added to the GRACE risk model [Net Reclassification Index (NRI) 41.2 (95% CI 12.0–70.4) *p* = 0.006].

**Conclusion:**

pDES concentrations predict clinical outcomes in patients with AMI, demonstrating its potential role as a prognostic marker in AMI.

## Introduction

Acute myocardial infarction (AMI) is the leading cause of mortality globally and is also associated with significantly higher morbidity (1). Current guidelines recommend formal early risk stratification in patients presenting with AMI (2). Currently, a number of scoring systems, such as the Global Registry of Acute Coronary Events (GRACE) score and the TIMI (Thrombolysis in Myocardial Infarction) risk score are recommended by clinical guidelines for assessment of the patient’s individual risk, to determine triage and aid management decisions (3, 4). Efforts have been made to develop stratifying biomarkers which can provide incremental prognostic information beyond these clinical risk scores. Currently, the most widely used biomarker for diagnosis of acute coronary syndromes (ACS) is the cardiac troponin. Initial cardiac troponin levels also provide prognostic information in terms of short- and long-term mortality, thus affecting a change in management of a patient with ACS (5). However, there is limited evidence regarding the optimal time points of serial troponin measurement. Also, Troponin is a marker of myocardial necrosis and not a specific marker of AMI. Similarly natriuretic peptides like B-type natriuretic peptide (BNP) and their precursor peptide N-terminal pro-BNP (NT-proBNP) are released in response to increased wall stress in the heart chambers. They also provide prognostic information in addition to cardiac troponin (6). Other biomarkers, such as high-sensitivity C-reactive protein, growth differentiation factor 15 (GDF-15), mid-regional pro-adrenomedullin, heart-type fatty acid-binding protein (h-FABP), copeptin, proenkephalin, and pro-substance P have also been shown to have some prognostic value (7). However, most of these biomarkers have, so far, not been shown to guide clinicians to the best management strategy in patients with AMI, and their added value in risk assessment beyond traditional risk factors and the GRACE risk calculation seems marginal.

Acute myocardial infarction is caused by vulnerable plaques. Plaque formation is a progressive inflammatory disease that leads to degradation and remodeling of the extracellular matrix, especially elastic fibers, and elastin (8). Since in AMI, plaque rupture invariably precedes cardiomyocyte damage, biomarkers of plaque instability in the form of elastin degradation products are logical candidates for AMI markers. Desmosine is the cross-link component in the elastin molecule and is released into its free form when elastin degradation occurs. Plasma desmosine (pDES) has been studied in independent clinical studies involving patients with diverse disease conditions ranging from patients with pulmonary conditions (9, 10), and atherosclerotic abdominal aortic aneurysm (11). In all these studies, pDES was strongly associated with cardiovascular outcomes. pDES thus has the potential to be a physiologically relevant biomarker of AMI. In this study, we sought to assess the predictive capacity of pDES for long-term adverse outcome in patients with AMI.

## Materials and methods

### Study participants

Overall, in our study we selected 236 patients with AMI divided into two group of patients that were sex- and age matched: 79 patients with AMI who died or had recurrent MI [death/myocardial infarction (MI)] at 2 years were compared to 157 patients with MI who did not have an event at 2 years. These patients were randomly selected from a cohort of 1,141 patients with AMI as described previously (12). In brief, these patients were admitted to University Hospitals of Leicester, UK, between August 2004 and April 2007 with a diagnosis of AMI. The original study involving human participants was reviewed and approved by the local ethics committee (Leicestershire, Northamptonshire, and Rutland Research Ethics Committee) and adhered to the Declaration of Helsinki. Each patient provided informed written consent to have blood samples stored for future research and outcomes surveyed. Diagnosis of AMI was based on cardiac troponin I (cTnI) concentration above the 99th percentile, along with at least one of the following: chest pain lasting >20 min or diagnostic serial electrocardiographic changes consisting of ST-segment and T-wave changes or new pathological Q-waves (13). Exclusion criteria included patients with malignancy, renal replacement therapy, or previous surgery within last 1 month. All participants received standard medical treatment for AMI and revascularization at the discretion of the attending physician. All these patients were all discharged from hospital. Patients with ample plasma sample volume from an original cohort of 1,141 patients were used in this study. Patients were then randomly selected using the SPSS random selection operation for age- and gender- matching for death/MI at 2 years and no event at 2 years groups.

### Sample processing and biomarker analysis

All blood samples were drawn at 3–5 days after hospital admission. Plasma samples were obtained by venipuncture, collected in pre-chilled tubes containing EDTA, and centrifuged at 1,500 × *g* for 20 min at 4^°^C and stored at −80^°^C. NT-proBNP was measured in all patients using a sandwich immunoassay as described previously (14). pDES levels were analyzed using a validated liquid chromatography-mass spectrometry/mass spectrometry (LC-MS/MS) as previously described (15). The lower limit of quantification is 0.1 ng/mL. pDES analyses were performed at the Biomarker and Drug Analysis Core Facility, Ninewells Hospital, University of Dundee, United Kingdom.

### Endpoints

Primary outcome for our study was a composite of all-cause mortality or reinfarction (death/MI) within 2 years. Outcomes of death/MI at 6 months and 1 year were also examined in regression analysis. Recurrent AMI was diagnosed using the universal definition (13). GRACE scores were calculated on hospital discharge for comparison with 6 months death and/or re-AMI. Addition of pDES to the GRACE score was tested for the end point of death/MI at 6 months. These outcomes of death/MI were obtained by reviewing the local hospital databases and the Office of National Statistics Registry, and also by telephone calls to patients, and those data were verified by reviewing medical records.

### Statistical analysis

Continuous data are presented as median (Q1–Q3). Categorical outcomes are given as absolute and relative frequencies (%). The continuous variables in the two independent groups were compared with the Mann–Whitney U test. Spearman’s rank correlations were calculated for pDES and clinical markers of cardiovascular disease (CVD). For regression analysis, all biomarker levels were log_10_ transformed. Logistic regression models were developed to calculate odds ratio (OR) of death/MI at 6 months, 1 year, and 2 years using log-transformed pDES values. Adjustments were made for traditional predictors of cardiovascular outcome in patients with AMI including age, sex, history of CVD, revascularisation, blood pressure, medications on discharge, Troponin I, NT-proBNP, and eGFR. Continuous reclassification analyses were used to assess the additional utility of pDES to the GRACE score for risk assessment at 6 months. Statistical analyses were performed using IBM SPSS Statistics (V26, IBM Corp., Armonk, NY, USA). A *p* < 0.05 was deemed statistically significant.

## Results

### Baseline characteristics

Clinical and laboratory baseline characteristics for the entire cohort and sub-cohorts split by the occurrence of death/MI at 2 years are shown in Table 1. Median age of the study population was 71 with a majority of male patients (67%). Patients with the composite outcome of death/MI had higher level of NT-proBNP compared to patients who did not have an event at 2 years (*p* = 0.016), and more likely to have Killip score > 1 (*p* = 0.013). There was no significant difference in the cTnI levels between the two groups. Table 2 shows clinical and laboratory baseline characteristics of the study cohorts split by median pDES levels.

**TABLE 1:** Study demographics for the total acute myocardial infarction (AMI) cohort and split according to outcomes of all-cause mortality or reinfarction at 2 years [death/myocardial infarction (MI)].

	Total cohort (*n* = 236)	No events at 2 years (*n* = 157)	Death/MI at 2 years (*n* = 79)	*P*-value
Age, years	71 (63–77)	70 (63–76)	71 (63–79)	0.368
Male	67%	68%	65%	0.663
History of MI/angina	29%	26%	35%	0.224
History of hypertension	53%	52%	57%	0.490
History of T2DM	23%	20%	28%	0.250
History of HF	7%	7%	6%	1.000
Killip class > 1	51%	46%	64%	0.013
Systolic blood pressure, mm Hg	128 (116–144)	128 (115–144)	130 (118–145)	0.891
Diastolic blood pressure, mm Hg	75 (70–84)	75 (68–82)	76 (71–86)	0.247
Heart rate, beats/min	74 (60–90)	71 (60–88)	76 (64–93)	0.109
Urea, mg/dL	6.2 (5.0–8.3)	5.9 (4.9–7.6)	7.2 (5.6–9.5)	<0.001
eGFR, ml/min/1.73 m^2^	66 (54–76)	68 (59–78)	59 (45–70)	<0.001
Sodium, mmol/L	137 (135–139)	137 (135–139)	137 (135–139)	0.409
Glucose, mmol/L	7.9 (6.5–10.2)	7.5 (6.3–9.9)	8.5 (7.1–11.8)	0.015
cTnI, ng/mL	6.4 (1.5–22.6)	6.2 (1.3–20.5)	7.2 (1.5–31.2)	0.544
NT-proBNP, pmol/L	1,707 (457–5,108)	1,471 (424–4,259)	3,078 (459–6,439)	0.016
STEMI patients	79%	79%	80%	1.000
Revascularisation	24%	19%	34%	0.010
ACEI on discharge	84%	90%	73%	0.002
Beta-blockers on discharge	83%	84%	80%	0.467
GRACE score	125 (109–145)	124 (108–139)	137 (109–159)	0.052
pDES level, ng/mL	0.40 (0.29–0.57)	0.36 (0.28–0.51)	0.45 (0.35–0.74)	0.002
pDES ≥ 0.4 ng/mL (number of patients)	118	70	48	0.013

*Data are presented as percentage (%) or median (interquartile range). ^†^ACEI, angiotensin converting enzyme inhibitor; cTnI, cardiac troponin I; eGFR, estimated glomerular filtration rate; HF, heart failure; MI, myocardial infarction; NT-proBNP, N-terminal pro-brain natriuretic peptide; pDES, plasma desmosine; STEMI, ST-segment elevation myocardial infarction; T2DM, type 2 diabetes mellitus.

**TABLE 2:** Study demographics for the total cohort and split by median plasma desmosine (pDES) levels.

	Total cohort (*n* = 236)	pDES < 0.4 ng/mL	pDES ≥ 0.4 ng/mL	*P*-value
Age, years	71 (63–77)	68 (63–74)	74 (64–79)	0.001
Male	67%	69%	64%	0.408
History of MI/Angina	29%	22%	36%	0.031
History of hypertension	53%	47%	59%	0.090
History of T2DM	23%	15%	31%	0.008
History of HF	7%	7%	7%	1.000
Killip class > 1	51%	41%	61%	0.003
Systolic blood pressure, mm Hg	128 (116–144)	130 (118–146)	125 (115–142)	0.281
Diastolic blood pressure, mmHg	75 (70–84)	76 (70–86)	75 (69–81)	0.362
Heart rate, beats/min	74 (60–90)	70 (60–92)	75 (61–88)	0.960
Urea, mg/Dl	6.2 (5.0–8.3)	5.8 (4.6–7.0)	6.8 (5.4–9.0)	<0.001
eGFR, ml/min/1.73 m^2^	66 (54–76)	68 (60–80)	62 (48–74)	<0.001
Sodium, mmol/L	137 (135–139)	137 (135–139)	137 (134–139)	0.335
Glucose, mmol/L	7.9 (6.5–10.2)	7.8 (6.5–9.7)	8.0 (6.4–11.8)	0.610
cTnI, ng/mL	6.4 (1.5–22.6)	7.3 (2.3–27.4)	4.4 (0.7–20.1)	0.025
NT-proBNP, pmol/L	1,707 (457–5,108)	1,116 (297–3,991)	2,408 (725–6,112)	<0.001
STEMI patients		86%	72%	0.01
Revascularisation		26%	21%	0.362
ACEI on discharge		89%	80%	0.072
Beta-blockers on discharge		87%	78%	0.085
GRACE score		122 (104–139)	134 (112–153)	0.006

*Data are presented as percentage (%) or median (interquartile range). ^†^ACEI, angiotensin converting enzyme inhibitor; cTnI, cardiac troponin I; eGFR, estimated glomerular filtration rate; HF, heart failure; MI, myocardial infarction; NT-proBNP, N-terminal pro-brain natriuretic peptide; pDES, plasma desmosine; STEMI, ST-segment elevation myocardial infarction; T2DM, type 2 diabetes mellitus.

### Correlations of plasma desmosine with clinical characteristics

Spearman’s rank correlation analyses showed that pDES levels correlated with renal markers – eGFR and urea (*r*_*s*_ −0.326 and 0.308, respectively, *p* < 0.001), age (*r*_*s*_ 0.293, *p* < 0.001), and the cardiac marker NT-proBNP (*r*_*s*_ 0.270, *p* < 0.001). Interestingly, pDES concentrations did not correlate with cTnI (*p* = 0.072) (Supplementary Table 1).

### Plasma desmosine and outcome of death/myocardial infarction

**FIGURE 1:**
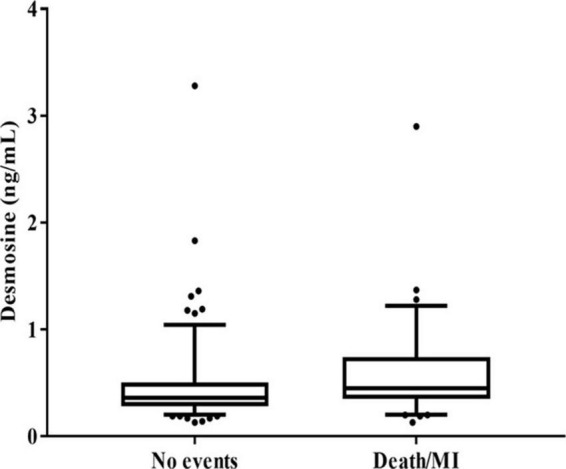
Box plot showing plasma desmosine (pDES) concentrations according to no events vs. death/myocardial infarction (MI) at 2 years (*p* = 0.002 by Mann–Whitney U test).

Median pDES of the study population was 0.4 ng/mL. Patients with the composite outcome of death/MI at 2 years had high pDES levels compared to those who did not have an event at 2 years (median levels 0.45 and 0.36 ng/mL, respectively *p* = 0.002) (Figure 1).

To further investigate association of pDES and outcome of death/MI at 6 months, 1 year, and 2 years, logistic regression analyses were conducted using multivariable models adjusted for independent predictors of outcome in AMI patients (Table 3). The univariable OR of pDES for the outcome of Death/MI at 2 years was 5.99 (95% CI 1.81–19.86, *p* = 0.003). In multivariable analysis for predicting Death/MI at 2 years, the ORs range from 6.29 (95% CI 1.78–22.18, *p* = 0.004) after adjustment for age, sex, past history of MI/angina in model 1, to 5.60 (95% CI 1.04–30.04, *p* = 0.044) in model 2 (adjusted for Age, sex, past history of MI/angina, Log-cTnI, systolic blood pressure, Log-NTproBNP, revascularisation, STEMI, β-blockers on discharge, and ACE/ARB on discharge). In model 3, addition of eGFR as a predictor revealed an OR of 8.07 (95% CI 1.08–60.45, *p* = 0.042) for the outcome of Death/MI at 6 months. However, for model 3, the OR was not significant as time of follow up increased.

**TABLE 3:** Logistic regression showing odds ratio (OR) for outcomes of death/myocardial infarction (MI) at 6 months, 1 year, and 2 years according to plasma desmosine (pDES) levels (unadjusted and adjusted for models 1, 2, and 3).

	Unadjusted			Adjusted for model 1^‡^			Adjusted for model 2^§^			Adjusted for model 3^#^		
				
	OR*	95% CI^†^	*P*-value	OR	95% CI	*P*-value	OR	95% CI	*P*-value	OR	95% CI	*P*-value
6 months	8.13	2.27–29.05	0.001	6.13	1.60–23.51	0.008	10.55	1.50–73.92	0.018	8.07	1.08–60.45	0.042
1 year	10.26	2.91–36.18	<0.001	8.78	2.34–32.91	0.001	8.69	1.45–52.09	0.018	6.34	0.99–40.70	0.052
2 years	5.99	1.81–19.86	0.003	6.29	1.78–22.18	0.004	5.60	1.04–30.04	0.044	3.57	0.62–20.74	0.156

*OD, odds ratio. ^†^CI, confidence interval. ^‡^Model 1: Age, sex, past history of MI/angina. ^§^Model 2: Age, sex, past history of MI/angina, Log-cTnI, systolic blood pressure, Log-NTproBNP, revascularisation, STEMI, β-blockers on discharge, ACEI/ARB on discharge. ^#^Model 3: Age, sex, past history of MI/angina, Log-cTnI, systolic blood pressure, Log-NTproBNP, revascularisation, STEMI, β-blockers on discharge, ACEI/ARB on discharge, eGFR.

The OR of pDES for the outcome of Death/MI at 6 months after adjustment for GRACE score was 5.23 (95% CI 1.37–19.91, *p* = 0.015) (Supplementary Table 2). Cox regression analyses were also conducted using multivariable models which also suggested that pDES is significantly associated with outcome across the three time points (Supplementary Table 3). Supplementary Figures 1, 2 show Kaplan–Meier survival analysis for outcomes of death/MI at 1 year and at 2 years based on median levels of pDES.

### Reclassification analysis

Reclassification analysis was performed using the net reclassification index (NRI) to assess the added value of pDES to the current GRACE clinical risk score for outcome of dead/MI at 6 months (61 events). Results showed that pDES was able to successfully down-classify risk in patients without an event at 6 months [NRI 26.4 (95% CI 11.6–41.3) *p* < 0.001], but unable to up-classify those with an event at 6 months [NRI 14.8 (95% CI −10.3–39.8) *p* = 0.249]. pDES, when added to the GRACE score, showed a total improvement in reclassification. [NRI 41.2 (95% CI 12.0–70.4) *p* = 0.006] (Table 4).

**TABLE 4:** Net reclassification index (NRI) for outcomes of death/myocardial infarction (MI) at 6 months adding plasma desmosine (pDES) to the global registry of acute coronary events (GRACE) clinical risk score.

	NRI	95% CI	*P*-value
Without endpoint	26.4	11.6–41.3	0.000
With endpoint	14.8	−10.3–39.8	0.249
Total	41.2	12.0–70.4	0.006

## Discussion

**FIGURE 2:**
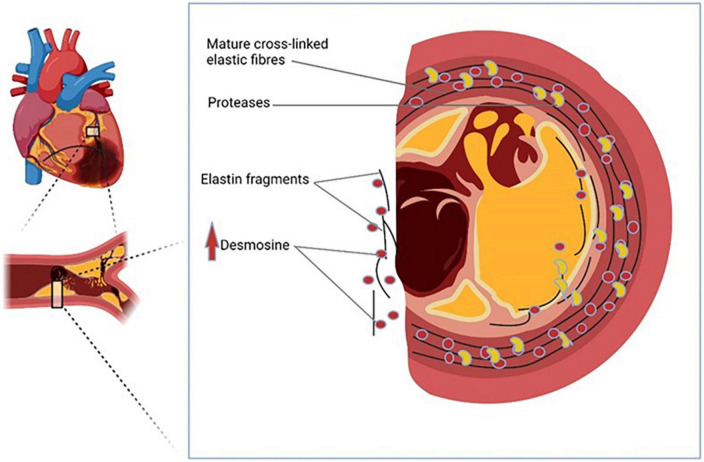
Plasma desmosine (pDES) is a potential novel biomarker of acute myocardial infarction (AMI).

Our study is the first report investigating the prognostic potential of pDES in a cohort of AMI patients from a single center. There are several novel findings in our study: Firstly, we have shown that in patients with AMI, pDES levels were higher in those who have poor outcome at 2 years. Secondly, pDES was associated with outcomes of death/MI at 6 months, at 1 year, and at 2 years even following multivariable adjustment for traditional predictors. Though in another multivariable model including eGFR, pDES was associated with the outcome only at 6 months follow-up, and less so as time of follow up increased. Finally, pDES was also able to reclassify risk stratification when added to the GRACE risk model for outcomes of death/MI at 6 months. These findings suggest that pDES can give additive prognostic information. pDES may therefore be a potential future biomarker for prediction of outcomes in patients with AMI (Figure 2).

Correlation analysis suggests that there was a positive relationship between pDES levels and age. These findings are in keeping with the results of previous studies since elastin degradation is accelerated during aging (9, 16). Though there was also a negative correlation between pDES and eGFR, this doesn’t seem to be clinically relevant as majority of the patients in our study had normal kidney function. Interestingly infarct size (as measured by troponin levels) were not correlated with pDES.

Data from our study indicated that pDES was associated with adverse cardiovascular outcomes including death and reinfarction. Many studies suggest that existing biomarkers such as troponin and NT-proBNP have a strong predictive value for mortality and heart failure, but show poor performance for association with reinfarction. This association of pDES in AMI with poor outcomes of death/MI may reflect a direct link between pathophysiological role of elastin degradation in atherosclerotic plaque progression and plaque instability. In fact, in a recent study, Van der Donckt et al. (17) utilized a unique mouse model of human end-stage atherosclerosis and plaque rupture with human-like complications to demonstrate the importance of elastin fragmentation in plaque instability and rupture. Measuring the breakdown products of elastin in the circulation therefore represents a logical means for the determination of atherosclerotic plaque instability. In our laboratory, we have developed a highly specific assay for analysis of pDES (15). Our group has previously explored the relationship between pDES and clinical outcomes in independent clinical studies. These studies have primarily focused on patients with pulmonary conditions, also demonstrated that pDES levels were elevated in patients with CVD, in particular in those with HF, MI, and hypertension, and was associated with CVD risk and mortality (9–11). Observations from these studies suggest that pDES predominantly reflects elastin degradation in the vascular tissue of the cardiovascular system, likely to be caused by vascular tissue inflammation and atherosclerosis. This may be one explanation for the role of pDES to reflect worsening cardiovascular outcomes and mortality. Our present study further advances these interesting exploratory findings from the above studies by investigating the role of pDES in the risk assessment of patients with AMI, and demonstrating its incremental prognostic value over and above the established risk factors and GRACE score. In our study, AMI patients with worse outcomes had high pDES levels. Also the combination of pDES and the GRACE risk score provided an improvement of reclassification for outcomes of death/MI at 6 months than the GRACE score alone, predominantly by down classifying those who did not have poor outcomes.

Risk stratification in patients with AMI is beneficial to estimate the prognosis. Due to new therapeutic approaches, this is important since it may help clinicians to select treatment regimens. A multimarker strategy using independent biomarkers might provide substantially more information for risk stratification since each marker reflects different pathobiological axes of response post-MI (18). Our study suggests that pDES can provide complementary information in risk stratification. Findings from our study are hypothesis generating for further investigating the combination of pDES and biomarkers of myocardial injury in a multimarker risk stratification. Future larger studies are needed to validate optimal cutoff thresholds and to investigate whether clinical use of pDES can improve therapeutic decision making in AMI patients.

### Limitations

This study has several limitations. First, it was a single-center study with a small sample size limited to only 236 MI patients because these 236 MI patients had the outcomes that were age and sex-matched. Therefore, the findings will need to be validated in another large cohort. Second, because of the case-control design of the study we cannot conclude whether pDES level is causally related to the development of cardiovascular events. Third this study included AMI patients from a historical cohort with a low rate of early revascularization unlike the current standard of care. However, the regression models of pDES with adverse events have been corrected for revascularization rate. Also, our study cohort did include patients with severe renal impairment end stage renal disease on dialysis. The risk predictive value of pDES in these patients are not known. Finally, the samples used in this current study was stored at −80^°^C for 15 years and the effect of such long-term storage to pDES analysis is difficult to evaluate. Our existing data indicate that it is at least stable at −80^°^C for 5 years. pDES is considered highly stable given the fact that it can sustain 108^°^C in strong acid for more than 20 h (19).

## Conclusion

In conclusion, we have found that pDES levels are associated with adverse clinical outcomes independent of established conventional risk factors and the commonly used biomarkers NT-proBNP and troponin. Furthermore, pDES provides incremental prognostic information beyond that provided by the GRACE score. Additional larger prospective studies are warranted to further evaluate clinical applicability of pDES as a prognostic biomarker in AMI.

## Data availability statement

The raw data supporting the conclusions of this article will be made available by the authors, without undue reservation.

## Ethics statement

The studies involving human participants were reviewed and approved by the Leicestershire, Northamptonshire, and Rutland Research Ethics Committee. The patients/participants provided their written informed consent to participate in this study.

## Author contributions

KA was involved in the initial writing of the draft of the manuscript and researching existing data on desmosine and biomarkers in Acute Myocardial Infarction. MZI was involved in completing the statistical analysis of the data. LLN provided the blood samples for the pDES analysis. IM and CCL were involved in reviewing and editing drafts of the manuscript. EK and JT-JH was involved in analyzing the plasma samples for desmosine and also in reviewing and editing drafts of the manuscript. A-MC was involved in reviewing and editing drafts of the manuscript and also oversaw the conduction of the project. A-MC, JT-JH, and LLN conceptualized the study. All authors contributed to the article and approved the submitted version.
