# Supplementary material for: Plasma desmosine for prediction of outcomes after acute myocardial infarction
Source: Front Cardiovasc Med. 2022 Nov 21;9:992388. doi: 10.3389/fcvm.2022.992388 (PMC9719937; doi:10.3389/fcvm.2022.992388)

**SUPPLEMENTARY**

**Supplementary Table 1.** pDES and correlations with clinical factors

|  | **r_s_** | ***p-*value** |
| --- | --- | --- |
| **eGFR** | -0.326 | <0.001 |
| **Urea** | 0.308 | <0.001 |
| **Age** | 0.293 | <0.001 |
| **NT-proBNP** | 0.270 | <0.001 |
| **cTnI** | -0.139 | 0.072 |
| **Diastolic BP** | -0.075 | 0.259 |
| **Systolic BP** | -0.068 | 0.309 |
| **Glucose** | 0.025 | 0.727 |
| **Heart rate** | 0.007 | 0.920 |
| **Sodium** | 0.004 | 0.958 |

**Supplementary Table 2.** Logistic regression showing odds ratio for outcomes of death/MI at 6 months according to pDES levels after adjustment for GRACE score

| **Adjusted for GRACE** | | | |
| --- | --- | --- | --- |
|  | **OR** | **95% CI** | **p Value** |
| **6 months** | 5.23 | 1.37-19.91 | 0.015 |

**Supplementary Table 3.** Independent prediction abilities of for log(pDES) using multivariable Cox survival analyses for outcomes of death/MI at 6 months, 1 year and 2 years

|  | **Unadjusted** | | | **Adjusted for model 1^‡^** | | | **Adjusted for model 2**^§^ | | | **Adjusted for model 3**^#^ | | |
| --- | --- | --- | --- | --- | --- | --- | --- | --- | --- | --- | --- | --- |
|  | **HR** | **95% CI** | **p Value** | **HR** | **95% CI** | **p Value** | **HR** | **95% CI** | **p Value** | **HR** | **95% CI** | **p Value** |
| 6 months | 5.90 | 2.24-15.51 | <0.001 | 4.67 | 1.66-13.15 | 0.004 | 4.65 | 1.27-17.02 | 0.020 | 5.56 | 1.20-25.75 | 0.028 |
| 1 year | 6.31 | 2.58-15.43 | <0.001 | 5.31 | 2.06-13.68 | 0.001 | 5.91 | 1.34-26.00 | 0.019 | 4.04 | 1.06-15.46 | 0.041 |
| 2 years | 4.49 | 1.88-10.70 | 0.001 | 4.32 | 1.74-10.71 | 0.002 | 3.58 | 1.05-12.17 | 0.041 | 3.15 | 0.88-11.26 | 0.078 |

**^‡^**model 1: Age, sex, past history of MI/angina

^§^model 2: Age, sex, past history of MI/angina, logTroponin, systolic blood pressure, LogNTproBNP, revascularisation, STEMI, β-blockers on discharge, ACE/ARB on discharge

#model 3: Age, sex, past history of MI/angina, Log-cTnI, systolic blood pressure, Log-NTproBNP, revascularisation, STEMI, β-blockers on discharge, ACE/ARB on discharge, log(eGFR)

Supplementary Figure 1. Kaplan-Meier survival curves for pDES levels at the median (0.4ng/mL) and outcomes of death/MI at 1 year (A) and at 2 years (B)


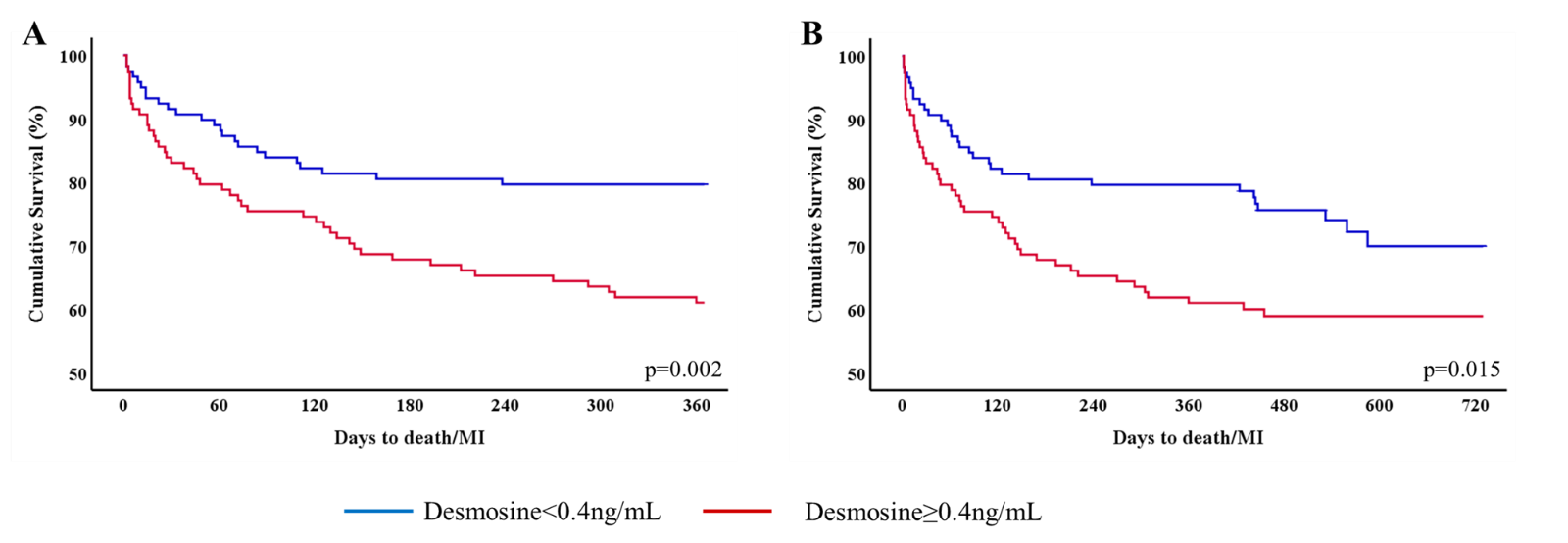


Supplementary Figure 2. Kaplan-Meier survival curves for desmosine levels at the median (0.4ng/mL) and outcomes of MI at 1 year (A) and at 2 years (B) and death at 1year (C) and at 2 years (D)


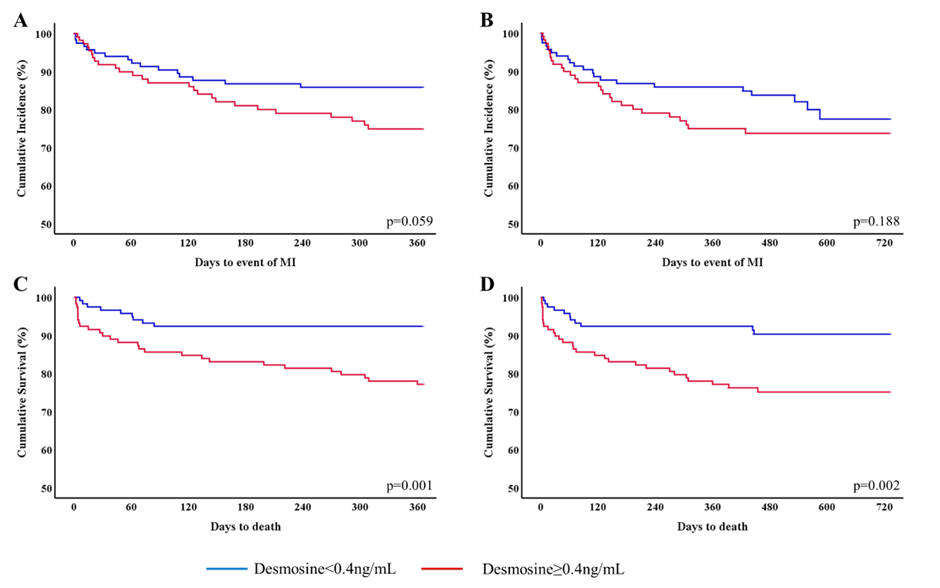

Supplement: Supplementary file 1 [file Data_Sheet_1.docx]
